# Identification and validation of cortisol‐related hub biomarkers and the related pathogenesis of biomarkers in Ischemic Stroke

**DOI:** 10.1002/brb3.3358

**Published:** 2024-01-29

**Authors:** Jing‐Jing Wang, Fang‐Biao Xu, Sen Hu, Yu‐Ming Xu, Xin‐Zhi Wang

**Affiliations:** ^1^ Neurology Department The First Affiliated Hospital of Zhengzhou University Zhengzhou China; ^2^ Neurology Department People's Hospital of Luanchuan Luoyang China; ^3^ Department of Encephalopathy The First Affiliated Hospital of Henan University of Chinese Medicine Zhengzhou China; ^4^ The First Clinical Medical College Henan University of Chinese Medicine Zhengzhou China; ^5^ Department of Medical Records Zhengzhou University People's Hospital Henan Provincial People's Hospital Zhengzhou Henan People's Republic of China

**Keywords:** bioinformatic analysis, biomarkers, cortisol, ischemic stroke, molecular mechanism

## Abstract

**Background:**

Ischemic stroke is a disease in which cerebral blood flow is blocked due to various reasons, leading to ischemia, hypoxia, softening, and even necrosis of brain tissues. The level of cortisol is related to the occurrence and progression of ischemic stroke. However, the mechanism governing their interrelationship is still unclear. The main objective of this study was to identify and understand the molecular mechanism between cortisol and IS.

**Methods:**

The common cortisol‐related biological processes were screened by mutual verification of two data sets and the cortisol‐related hub biomarkers were identified. Modular analysis of protein interaction networks was performed, and the differential pathway analysis of individual genes was conducted by GSVA and GSEA. Drug and transcription factor regulatory networks of hub genes were excavated, and the diagnostic potential of hub genes was analyzed followed by the construction of a diagnostic model.

**Results:**

By screening the two data sets by GSVA, three biological processes with common differences were obtained. After variation analysis, four cortisol‐related hub biomarkers (CYP1B1, CDKN2B, MEN1, and USP8) were selected. Through the modular analysis of the protein‐protein interaction network and double verification of GSVA and GSEA, a series of potential molecular mechanisms of hub genes were discovered followed by a series of drug regulatory networks and transcription factor regulatory networks. The hub biomarkers were found to have a high diagnostic value by ROC; thus, a diagnostic model with high diagnostic efficiency was constructed. The diagnostic value was mutually confirmed in the two data sets.

**Conclusion:**

Four cortisol‐related hub biomarkers are identified in this study, which provides new ideas for the key changes of cortisol during the occurrence of IS.

## Graphical abstract

In conclusion, This study is the first molecular study on cortisol‐related biological processes. The results can help us better understand the biological processes related to cortisol and its potential importance with other pathology in IS. Patients may get better curative effect by personalized treatment.

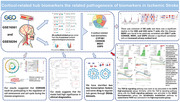

## 1 INTRODUCTION

Stroke is a life‐threatening event all across the globe and China is reported to be one of the most affected countries in the world. Based on the reports from the 2019 “Global Burden of Disease Study,” 3.94 million new cases, 28.76 million prevalent cases, and 2.19 million stroke fatalities were recorded in China in 2019. In China, stroke is also the main contributor to disability‐adjusted life years (DALYs). The total number of DALYs was 45.9 million in 2019. According to the data of 1672 public tertiary care hospitals recorded in the “Hospital Quality Monitoring System” (HQMS), there were 3,411,168 hospital admissions due to stroke in the year 2019, of which 281,875 cases (82.6%) were of ischemic strokes (IS). The rate of in‐hospital death/discharge was 6.0%, and the median length of hospital stay and the interquartile range (IQR) was 10.0 (7.0–13.0) days. Stroke, especially IS, is a huge threat to human health (Wang et al., 2022). The basic mechanism of IS involves the obstruction of blood flow to the brain parenchyma, resulting in local ischemia and hypoxia in the brain tissue (Herpich & Rincon, 2020).

**FIGURE 1:**
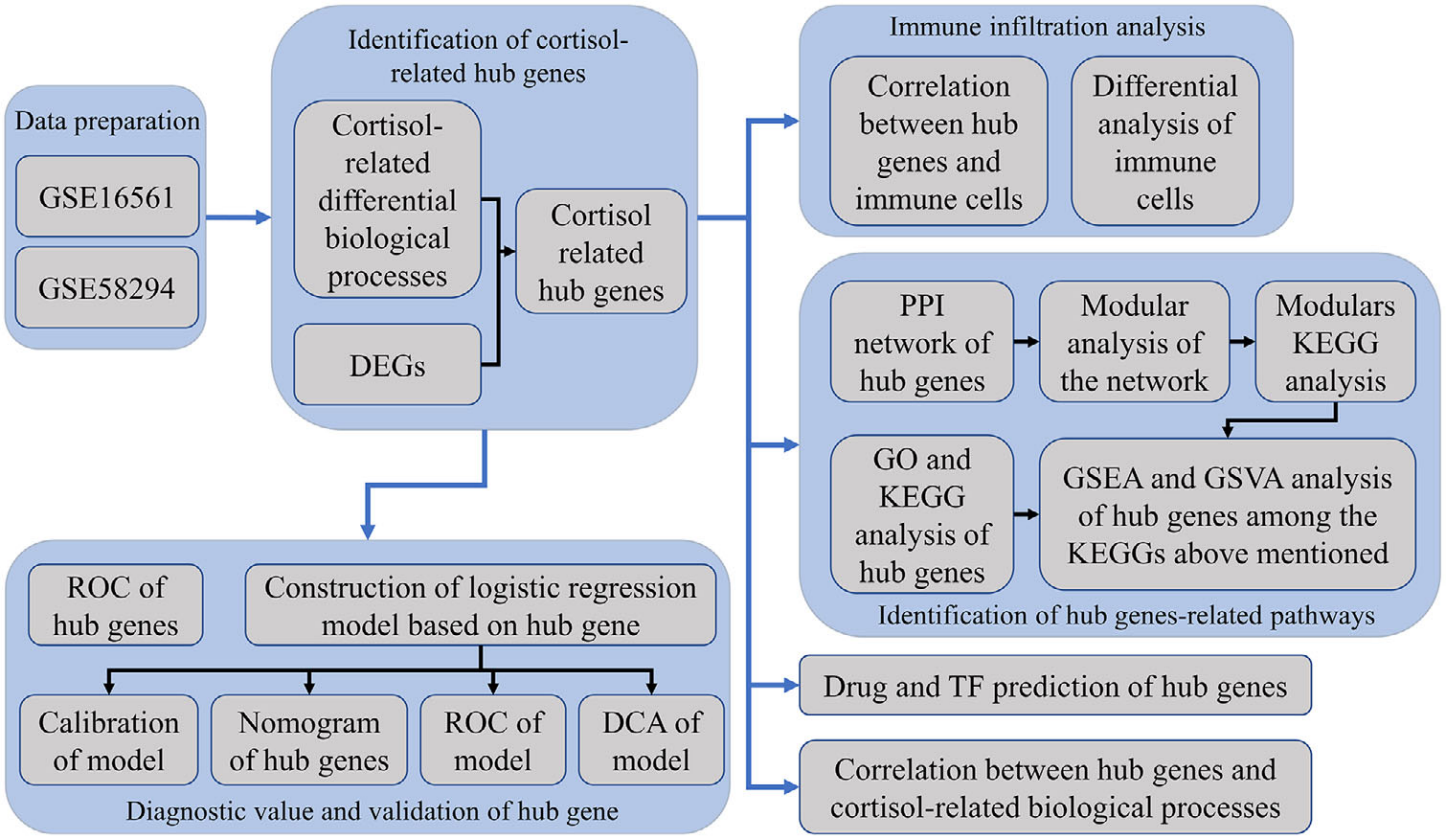
Flowchart of the study.

Elevated serum cortisol levels may lead to vascular atherosclerosis, which increases vascular resistance, slows down the blood flow, and increases the risk of cardiovascular and cerebrovascular diseases (Aresta et al., 2021). A systematic review showed that the cortisol level after stroke was closely associated with morbidity and mortality, and remained high or continued to rise for a week (Barugh et al., 2014). Several studies have confirmed that the levels of serum cortisol have independent associations with infection risk after a stroke, which is probably linked to the fact that serum cortisol levels are independent factors associated with neutropenia and lymphopenia (Tanzi et al., 2011; Zierath et al., 2018). These associations suggest that cortisol levels could be utilized for the prognosis of stroke patients. However, most clinical studies on strokes do not explain the molecular mechanism between cortisol and IS. Therefore, we attempted to identify and explore key genes between cortisol and IS and their potential pathways by analyzing the existing IS‐related data sets to provide a reference and a basis for cortisol level regulation, prevention, and improvement of IS prognosis. The above research has been represented as a roadmap (Figure 1).

## 2 METHODS

### 2.1 Data source and normalization

In this study, the keyword “strokes” was used for searching and screening. Two datasets GSE16561 and GSE58294 related to human peripheral blood were retrieved from the GEO database (https://www.ncbi.nlm.nih.gov/geo/). The GSE16561 dataset contained 39 IS and 24 healthy controls, and the GSE58294 dataset contained 23 control samples and 69 cardioembolic stroke samples. Using cortisol as the keyword, the MSigDB database (Subramanian et al., 2005) (https://www.gsea‐msigdb.org/gsea/msigdb) was searched to obtain cortisol‐related gene sets. The “normalizeBetweenArrays” function (Bolstad et al., 2003) of the package “limma” was used to perform log2 correction to the original count matrix.

### 2.2 Single sample gene set enrichment analysis

Three different R packages (GSVA (Hänzelmann et al., 2013), limma (Ritchie et al., 2015), and GSEABase (Morgan, 2022)) were used to execute single sample gene set enrichment analysis (ssGSEA) of the cortisol‐related gene sets belonging to each of the two data sets, and the differential analysis between the control group and the disease group was performed by wilcox.test; finally R package “ggplot2” was used to generate the violin plot (Villanueva & Chen, 2019). The gene set in the cortisol‐related biological process with significant differences in the data of the two groups was defined as the cortisol‐related gene set in this study.

### 2.3 Identification of differentially expressed genes

Differential analysis of the two data sets was performed by the R package “Limma” [10]. Probe sets that lacked a matching gene symbol were removed. Averaging was done for genes with > 1 probe set. Genes with a *p* value of < .05 and a |fold change| of ≥1 were considered to be differential genes.

### 2.4 Identification of cortisol‐related hub genes

The R package “VennDiagram” (Chen & Boutros, 2011) was used to analyze and draw Venn diagrams between the two data sets and the cortisol‐related gene sets; the intersection genes were defined as the key cortisol‐related genes. In the context of the GSE16561 dataset, Spearman (Wissler, 1905) was employed to examine the association between the hub genes and the cortisol‐related biological processes.

### 2.5 Immune infiltration analysis

Using the GSE16561 data set and the CIBERSORT algorithm (Newman et al., 2015), which deconvoluted the expression matrix of human immune cell subtypes, it was possible to further analyze the immune microenvironment in the peripheral blood of IS patients. This analysis was based on the linear support vector regression principle. Further, the limma10 package was used to analyze the variations in immune cells between IS and the control group and Spearman (Wissler, 1905) was used to analyze the correlation between hub genes and immune cells.

### 2.6 Enrichment analyses of hub genes

The R package “clusterProfiler” (Yu et al., 2012) was used for Gene Ontology (GO) and Kyoto Encyclopedia of Genes and Genomes (KEGG) enrichment analysis of the hub genes. Both KEGG and GO are relatively large gene annotation databases. For example, KEGG can be used to analyze the signaling pathways that may be activated by genes based on existing gene sets and can effectively help analyze the potential molecular mechanisms of the genes.

### 2.7 Construction of protein‐protein interaction network and module analysis

The GeneMANIA (Warde‐Farley et al., 2010) (http://www.genemania.org/) database was used for the construction of a protein‐protein interaction network with the hub genes. The protein‐protein interaction of the submitted gene sets could be obtained through the database, which was not limited to their internal protein‐protein interactions. To ensure the authenticity of the interactions, all interactions containing nondifferential genes were eliminated in this study. Further, the “walktrap.community” algorithm in the R package “igraph” (Csardi & Tamas, 2006) was used to conduct modular analysis of the interaction network, and KEGG enrichment analysis was performed for each of the modules to analyze the key pathways of the hub genes (Saini et al., 2017).

### 2.8 Gene set enrichment analysis (GSEA) and gene set variation analysis (GSVA) of hub genes

To more accurately explore the differences in the signaling pathways activated in diseases due to the different expression levels of the hub genes, two expression groups were established based on the median expression, namely, a low‐expression group and a high‐expression group. All the pathways enriched by each module of the PPI network were used as the background for GSEA and GSVA enrichment analysis. Both GSEA and GSVA were based on the gene expression levels, and the difference in the activation degree of the signaling pathway between the two groups was obtained through calculation. To make a more accurate judgment, the two algorithms, GSEA and GSVA, were used to verify each other.

### 2.9 Diagnostic value and validation of hub genes

To further investigate the diagnostic value of the hub genes and to verify the diagnostic performance of the hub genes in the two data sets, the R package “pROC” (Robin et al., 2011) was used. To facilitate clinical application, a logistic regression‐based IS diagnostic model was developed, a nomogram was drawn, and the DCA and C index was used to verify the reliability of the model.

### 2.10 Drug and transcription factors (TF) prediction of the hub genes

To study the regulation of the hub genes, TFs of hub genes were enriched through the TRRUST online database (Han et al., 2018) (https://www.grnpedia.org/trust/) and verified by dataset differential analysis. Through text mining of the TRRUST online database, a large number of TF regulation information was collected, screened, and sorted manually. The prediction results had a high reference value. The DGIdb online database (Freshour et al., 2021) (https://dgidb.genome.wustl.edu/) was used to predict drugs that could regulate the hub genes. The DGIdb database incorporated multiple drug‐gene interaction databases, with abundant and authentic information. Finally, all the information was imported into “Cytoscape” software (Shannon et al., 2003) and network regulation maps were constructed.

## 3 RESULTS

### 3.1 Single sample gene set enrichment analysis in GEO datasets

**FIGURE 2:**
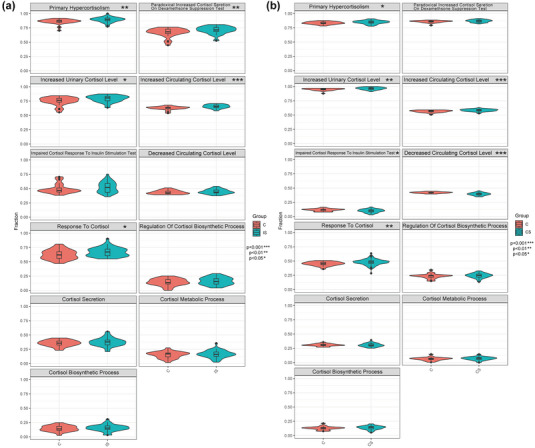
Differential analysis of cortisol‐related biological processes between the disease group and the healthy control group. (a) GSE16561 and (b) GSE58294.

By searching the MSigDB database, 11 cortisol‐related biological processes were obtained. The ssGSEA algorithm was then used to analyze the variations between the control and the disease group of the two data sets (GSE16561 and GSE58294) (Figure 2). There was a significant difference in the primary hypercortisolism, increased circulating cortisol level, increased urinary cortisol level, and cortisol response. So, the genes contained in these four biological processes were used as cortisol‐related genes, with a total of 26 genes (Table 1).

**TABLE 1:** Genes of cortisol‐related biological processes.

Name	Sources	Genes
HP_PRIMARY_HYPERCORTISOLISM	http://www.gsea‐msigdb.org/gsea/msigdb/human/geneset/HP_PRIMARY_HYPERCORTISOLISM	CDKN2B PRKACA PRKAR1A CDKN1B CDKN2C CDKN1A MEN1 GNAS PDE11A
HP_INCREASED_URINARY_CORTISOL_LEVEL	http://www.gsea‐msigdb.org/gsea/msigdb/human/geneset/HP_INCREASED_URINARY_CORTISOL_LEVEL	TP53 PRKAR1A PRKACA CDH23 PDE11A CDKN1B PDE8B NR3C1 ARMC5 USP8 GNAS
HP_INCREASED_CIRCULATING_CORTISOL_LEVEL	http://www.gsea‐msigdb.org/gsea/msigdb/human/geneset/HP_INCREASED_CIRCULATING_CORTISOL_LEVEL	CYP11B2 TP53 PRKAR1A PRKACA GNAS KCNJ11 PDE11A MEN1 ARMC5 HTR1A CDKN2B CDH23 CDKN1B AIRE PDE8B USP8 CDKN2C NR3C1 CYP11B1 RET CDKN1A
GOBP_RESPONSE_TO_CORTISOL	http://www.gsea‐msigdb.org/gsea/msigdb/human/geneset/GOBP_RESPONSE_TO_CORTISOL	CYP1B1 IGFBP7 SLIT3 KLF9 SLIT2

### 3.2 Identification of differentially expressed genes in GSE16561 and GSE58294 expression profiles

**FIGURE 3:**
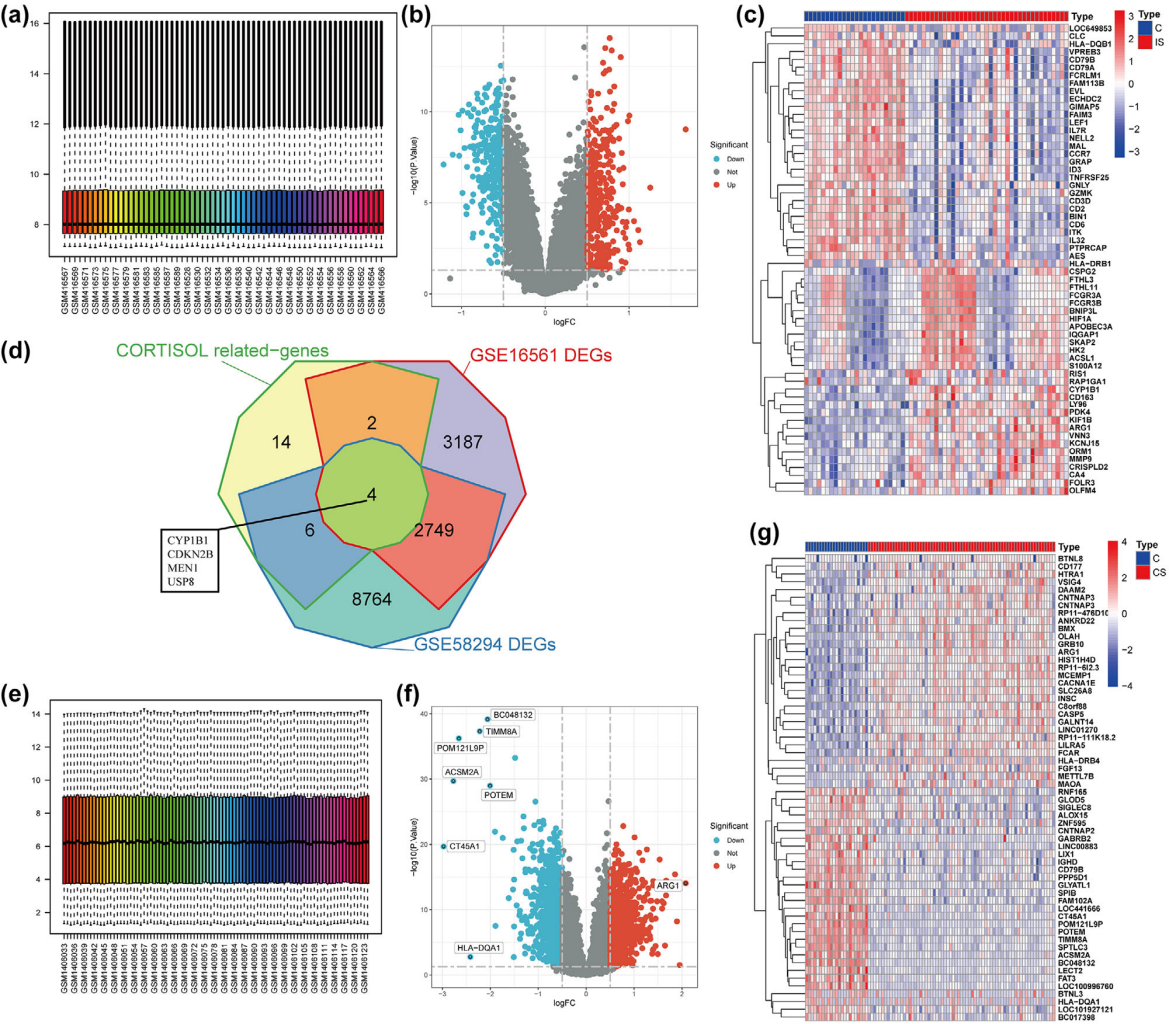
(a) Gene expression of each sample after GSE16561 gene expression matrix normalized by log2. (b) The GSE16561 dataset differential gene volcano plot. (c) The volcano plot of GSE16561 dataset with the top 30 gene expression difference. (d) VEEN plot of the cortisol‐related gene set and GSE16561, GSE58294 data set differential genes. (e) Gene expression box plot of each sample after GSE58294 gene expression matrix normalized by log2. (f) Differential gene volcano map in GSE58294 data set. (g) The volcano plot of GSE58294 dataset with the top 30 gene expression difference.

Log2 normalization was adopted for processing the raw data. The expression level information of the processed samples is shown (Figure 3a and e). Volcano maps constructed from the differential analysis of the two datasets are shown (Figure 3b and f). Heat maps of the top 30 genes with the largest differences are presented (Figure 3c and g). The DEGs of the two data sets were then intersected with the 26 cortisol‐related genes obtained in the previous step to get the four cortisol‐related hub genes (CYP1B1, CDKN2B, MEN1, and USP8) as shown (Figure 3d).

### 3.3 Enrichment analysis of the hub genes

**FIGURE 4:**
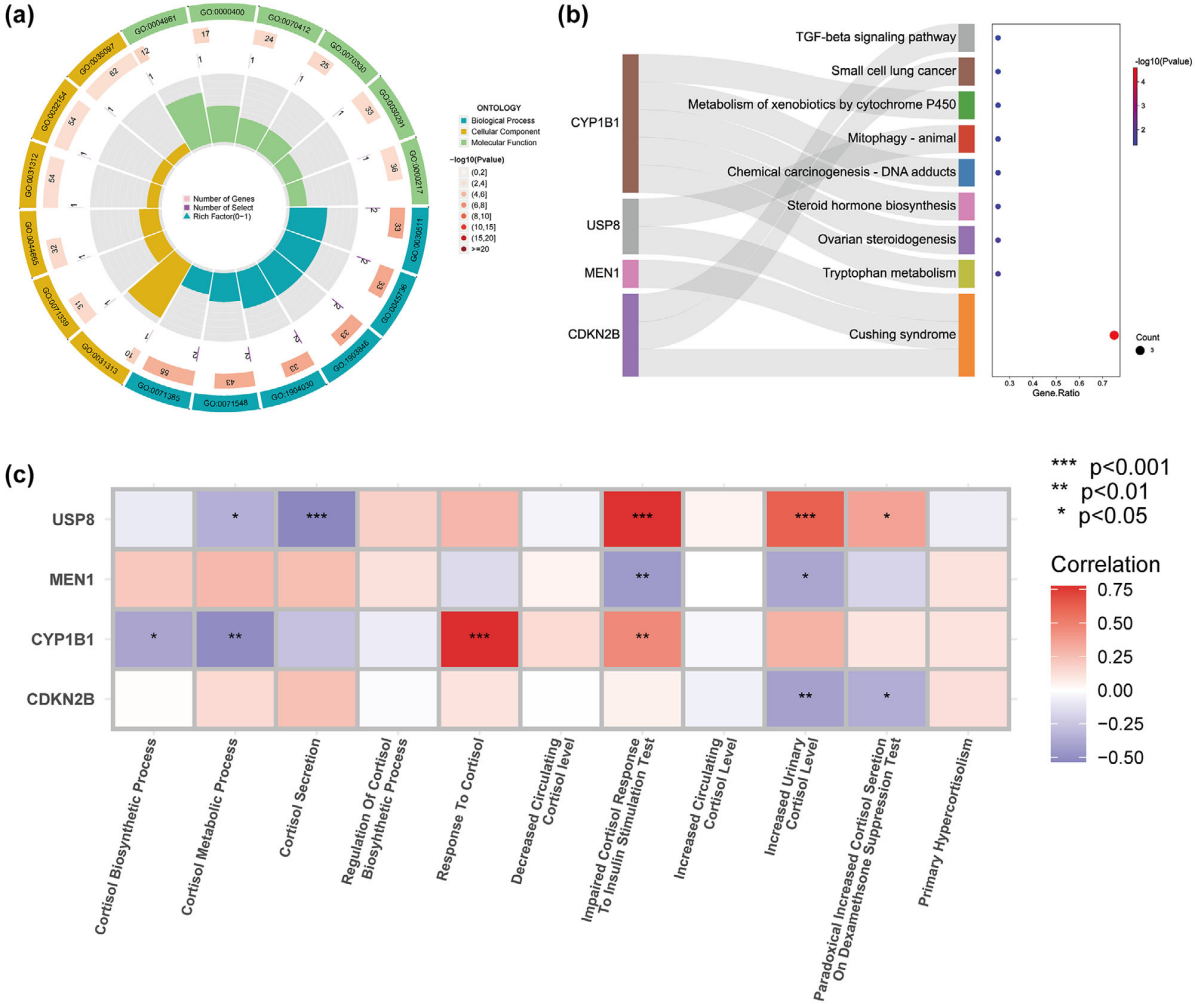
(a) GO enrichment circle diagram of cortisol‐related hub genes. (b) KEGG enrichment Sankey diagram and bubble plots of cortisol‐related hub genes. (c) Correlation heat map of hub genes and cortisol‐related biological processes.

GO circular diagram (Figure 4a) was constructed based on the GO & KEGG enrichment analysis performed on the hub genes; a bubble diagram combination (Figure 4b) was generated based on the KEGG enrichment genes and pathway Sankey diagram combined with pathway enrichment. The biological processes of GO enrichment were mainly enriched in the regulation of cell cycle‐dependent proteins and the cellular response to glucocorticoids and corticosteroids. The KEGG pathway was significantly enriched in Cushing syndrome. From the enrichment results of the two aspects, it was not difficult to infer that there was a significant association between the hub genes and the regulation of cortisol.

### 3.4 Association of candidate genes with immune cell infiltration and cortisol‐related biological processes

**FIGURE 5:**
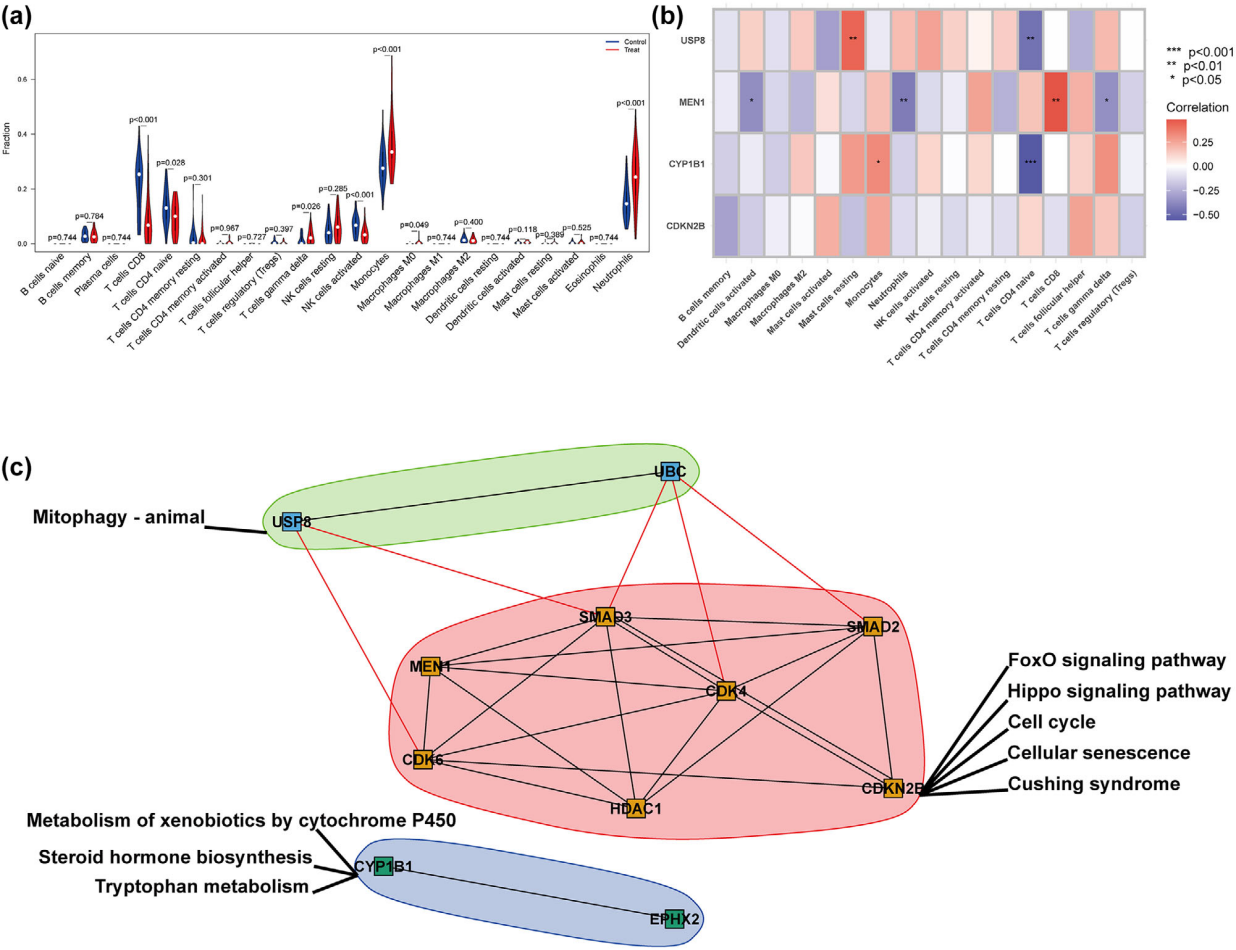
(a) GSE16561 data set violin plot of the difference between the immune cell disease and the healthy control group after calculation by the CIBERSORT algorithm. (b) Heat map of the correlation of immune cell content between hub genes and the disease group. (c) Protein interaction network and modular KEGG enrichment network diagram after Walktrap.community algorithm modular analysis.

The immune cell infiltration was estimated using the CIBERSORT algorithm that used the gene expression matrix of the GSE16561 dataset, and the difference between IS and healthy controls was determined, as shown (Figure 5a). As can be seen (Figure 5b), a correlation between the hub genes and the immune cell content was calculated. Also, a correlation between the hub genes and the biological processes connected to cortisol was determined (Figure 4c). The results revealed that in comparison to the healthy control group, there was activation of NK cells and there was a significant decline in the CD8 and CD4 naïve T cells after the disease, which suggested the weakening of the body's immunity; Monocytes, macrophages M0, and neutrophils were elevated significantly, suggesting the continuation of inflammatory responses. Among these differential immune cells, MEN1 was found to be negatively correlated with the number of neutrophils and positively correlated with CD8 T cells. As per our observations, none of the four hub genes had a significant correlation with primary hypercortisolism and increased circulating cortisol levels. Only CDKN2B, MEN1, and USP8 showed a significant correlation with an increased urinary cortisol level; only CDKN2B and MEN1 showed a significant negative correlation with an increased urinary cortisol level, suggesting that CDKN2B and MEN1 may have inhibitory effects on the elevation of nephrogenic cortisol.

### 3.5 Identification of hub genes‐related pathways

**FIGURE 6:**
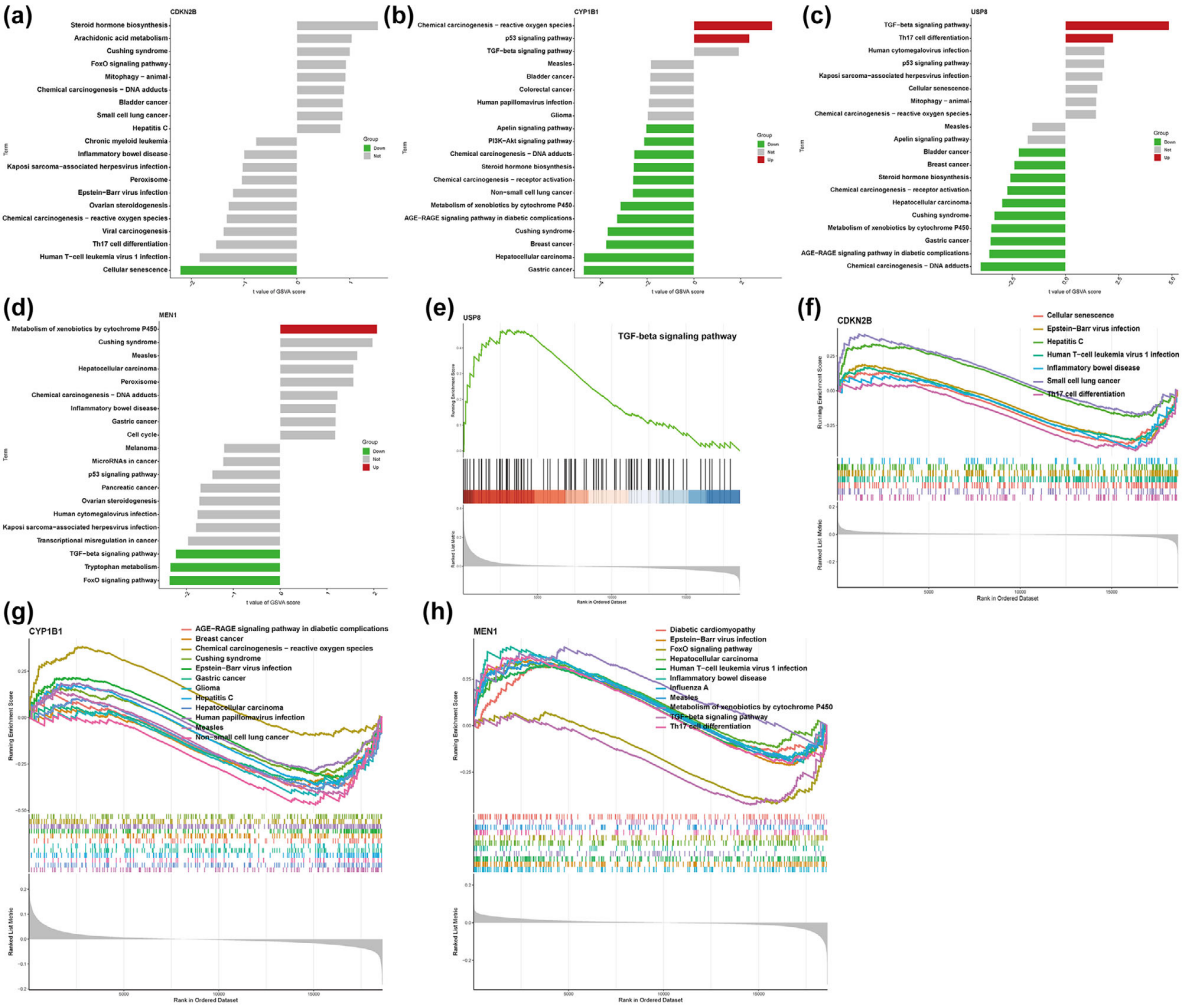
GSVA (a) and GSEA (f) analysis of CDKN2B in the disease group. GSVA (b) and GSEA (g) analysis of CYP1B1 in the disease group. GSVA (c) and GSEA (e) analysis of USP8 in the disease group. GSVA (d) and GSEA (h) analysis of MEN1 in the disease group.

Protein‐protein interactions related to the hub genes were obtained through GeneMANIA; the PPI networks were constructed after screening differential genes in the dataset GSE16561. The walktrap.community was used to perform modular analysis and enrichment of the KEGG pathways, as shown (Figure 5c). Our results suggested that CDKN2B could be participating in the regulation of cell senescence and cell cycle during the disease progression. To further confirm the signaling pathways involved with hub genes during the disease progression, GSEA and GSVA methods were used. We evaluate the activation of pathways in high‐ and low‐expression groups of the hub genes, as shown (Figure 6). Through mutual verification of the two algorithms, it was not difficult to deduce that the cellular senescence signaling pathway was activated in a CDKN2B low‐expression group. The chemical carcinogenesis‐reactive oxygen species signaling pathway was activated in a CYP1B1 high‐expression group, whereas some cancer pathways such as gastric cancer and Cushing syndrome were triggered in the low‐expression group. The TGF‐β signaling pathway was seen to be stimulated in the USP8 high‐expression group. Similarly, while the TGF‐β signaling pathway along with the FoxO signaling pathway was activated in the MEN1 low‐expression group, the xenobiotics metabolism pathway by cytochrome P450 was activated in the high‐expression group.

### 3.6 Diagnostic value and validation of hub gene

**FIGURE 7:**
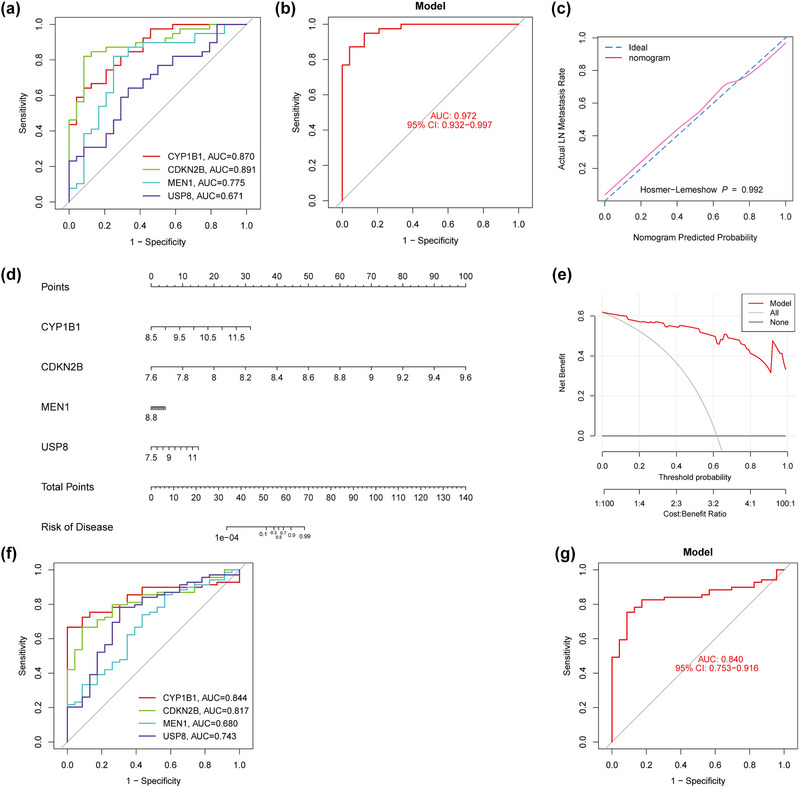
(a) ROC curve of hub genes in the GSE16561 dataset. (b) ROC curve of the logistic regression diagnostic model constructed by hub genes. (c) The calibration plot of the diagnostic model and the Hosmer‐Lemeshow test for the curve. (d) The nomogram of the diagnosis model. (e) DCA curve of the diagnostic model. (f) ROC curve of hub genes in the GSE58294 dataset. (g) Verification of the ROC curve of the logistic regression diagnostic model constructed by the hub genes in the GSE58294 dataset.

To further explore the diagnostic power of the hub genes, a ROC of the hub genes was calculated using two data sets, as shown (Figure 7a, GSE16561 and Figure 7f, GSE58294). It was observed that CYP1B1 and CDKN2B possessed high diagnostic efficiency in different data sets, and the logistic regression model was used to construct diagnostic models in the two data sets. The AUC values are shown (Figure 7b, GSE16561 and Figure 7g, GSE58294). Our results suggest that the disease diagnosis models constructed by the four hub genes had significantly high diagnostic efficiency. To facilitate clinical application, a nomogram was built, as shown (Figure 7d), and the diagnostic model was evaluated by calibration (Figure 7c) and DCA (Figure 7e). Calibration results confirmed that the degree of fitting between our model and the ideal model was high. Similarly, the *p* value obtained from the Hosmer‐Lemeshow test was 0.992 (>0.05), indicating no significant difference between the true value and the predicted value. DCA decision curve indicated that when the risk threshold (Pi) was 1.0, the diagnostic model still had a high net benefit rate (NB), suggesting that the model had high significance in clinical diagnostics.

### 3.7 Drug and TF prediction of the hub genes

**FIGURE 8:**
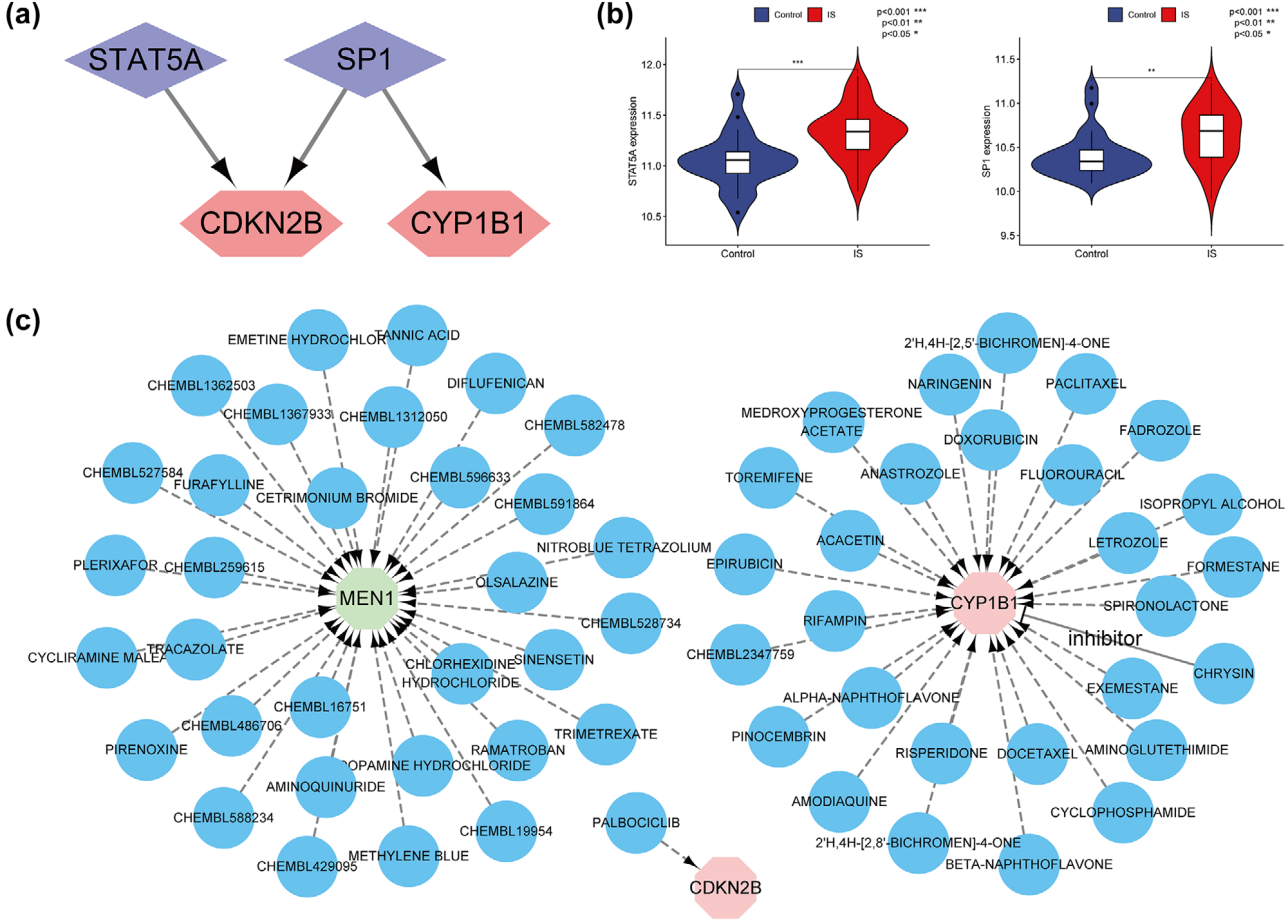
(a) TF‐mRNA regulatory network. (b) Differential analysis boxplot of TF in GSE16561 dataset. (c) Drug‐mRNA regulatory network.

Transcription factors of the hub genes were predicted through online databases and verified by differential analysis. It shows the regulatory network of TF genes (Figure 8a) and the difference box plot is shown (Figure 8b). In addition, small drug molecules that can regulate hub genes were predicted using the drug database, and a drug‐genes network diagram was constructed (Figure 8c), which provides a foundation for subsequent research on transcriptional and drug regulations.

## 4 DISCUSSION

The correlation between cortisol levels and cardiovascular/cerebrovascular diseases has been confirmed in many studies; salivary cortisol has been used as a predictor of cognitive impairment after stroke (Wang et al., 2021). In this study, through a series of bioinformatics analyses and verification of additional data sets, four key genes (CYP1B1, CDKN2B, MEN1, and USP8) in cortisol‐related biological processes were obtained; their diagnostic value, affected pathways in the disease process, and correlation with immune infiltration, etc. were also analyzed.

We discovered a total of four biological processes with a significant difference in the disease group compared to the control group, which includes primary hypercortisolism, increased circulating cortisol level, increased urinary cortisol level, and cortisol response. Among these, serum cortisol and urinary cortisol showed a significant increase in the disease group, which is consistent with existing findings. Studies (Chen et al., 2022; Olsson, 1990) have shown that urinary cortisol had a significant positive correlation with poor prognosis of stroke. In the current study, urinary cortisol level was found to have a significant positive correlation with USP8, but a significant negative correlation with MEN1 and CDKN2B. As was found in a clinical cohort study, Cushing disease patients with USP8 mutations had higher urinary cortisol levels and were more likely to relapse after treatment (Albani et al., 2018); our results support this finding. However, the association of MEN1 and CDKN2B with urinary cortisol levels is yet to be described. Another study (Miró‐Mur et al., 2018) showed that serum cortisol was significantly correlated with enhanced infarct growth, which in turn was linked to the reduction of circulating lymphocytes. In this study, the algorithm suggested that NK cells were activated, while CD8 and CD4 naïve T cells decreased significantly as a disease response, which is consistent with findings from previous studies. Interestingly, in this study, a significant negative correlation was observed between USP8‐CYP1B1 and CD4 naïve T cells; a significant positive correlation was detected between MEN1 and CD8 T cells. Perhaps USP8 and MEN1 are the key genes between cortisol regulation and lymphopenia, which needs further confirmation.

The protein translated by CYP1B1 is Cytochrome P450 1B1, a kind of cytochrome in human cells. The growing evidence suggests that CYP1B1 or its genetic variation may be associated with the risk of IS. A case‐control study identified two CYP1B1 gene variants (rs10916 and rs2855658) that are associated with increased risk of IS in the Chinese population. This provides valuable insights for the individualized prevention and treatment of IS. This is an important finding, as it may help us better understand the genetic mechanisms of this disease and lead to new therapeutic strategies. (Zhang et al., 2023). It has been demonstrated previously that this protein has involvement in the metabolism of various endogenous substrates such as arachidonic acid in fatty acids (Mesaros et al., 2010), estradiol in steroid hormones (Badawi et al., 2001), and so on. Studies have shown that in the pulmonary blood vessels, estradiol was metabolized by CYP1B1, and antivascular endothelial cells (Tofovic, 2010) produced 2‐methoxy estradiol. However, estradiol has been proven to significantly improve the peripheral immunosuppression caused by stroke in female stroke mice, thereby reducing the stroke area (Zhang et al., 2010). So, it is believed that CYP1B1 is a key protein that regulates estradiol levels after stroke. The signaling pathways for steroid hormone biosynthesis and the metabolism of xenobiotics by cytochrome P450 were both enriched in our analysis of the enrichment of four hub genes and the pathway enrichment of protein interaction modular analysis screened by differential analysis in the data set. Two such signaling pathways may be the key pathways that got activated during the occurrence and progression of the disease. Meanwhile, in this study, CYP1B1 was significantly related to the biological process response to cortisol, and such biological processes were significantly different between the diseased and healthy controls, suggesting that CYP1B1 may regulate the body's response to cortisol through all metabolic pathways of endogenous substrates, which in turn affects the incidence and progression of the disease. After the onset of the disease, according to the calculation of GSVA and GSEA, underexpression of CYP1B1 would activate the Cushing syndrome pathway, and overexpression of CYP1B1 will activate chemical carcinogenesis‐reactive oxygen species. Based on the results of immune cell infiltration, CYP1B1 may be involved in the reactive oxygen pathway to change the production of reactive oxygen species, thereby affecting the change in the number of CD4 naïve T cells after the onset of the disease. At present, it is known that reactive oxygen species are closely linked to CD4 T cells (Lin et al., 2021), which opens avenues for further study. To further explore the connection between transcriptional regulation and drug regulation of hub genes, the CYP1B1 transcription factors and corresponding regulatory drugs were screened via differential analysis of databases and data sets. Prior experimental data suggest that inhibiting the expression of SP1 can reduce the expression of CYP1B1 significantly, thereby reducing the occurrence of cell proliferation, metastasis, and carcinogenesis (Tsuchiya et al., 2003). In drug screening, CHRYSIN is found to inhibit the expression of CYP1B1, which is worthy of future investigations.

USP8 (Ubiquitin carboxyl‐terminal hydrolase 8) is a kind of deubiquitinating enzyme. And it is a gene encoding ubiquitin carboxyl‐terminal hydrolase 8 protein. A research has found that USP8 is mainly expressed in microglia (IBA1 positive cells), and its expression significantly decreases after LPS treatment. This leads to the activation of the TLR4/MyD88/NF‐κB signaling pathway that should have been suppressed, exacerbating the hippocampal damage and behavioral defects caused by LPS in mice (Zhao et al., 2020). Studies have shown that in Parkinson's disease, inhibiting the expression of USP8 would lead to a delay in the occurrence of mitophagy and a reduced success rate (Durcan & Fon, 2015), thereby affecting the quality control process of mitochondria. In this study, through the dual screening of GSVA and GSEA, it was found that in the disease group, the high expression of USP8 activated the TGF‐β signaling pathway. Another ovarian cancer experimental study (Jin et al., 2020) showed that the overexpression of TGF‐β promotes the occurrence of mitophagy. In this study, the expression of USP8 was found to have a significant negative correlation with the CD4 T cells in the disease group. In summary, high expression of USP8 may promote the occurrence of mitophagy through the TGF‐β signaling pathway, thereby reducing the number of CD4 naïve T cells, which needs further confirmation.

CDKN2B (Cyclin‐dependent kinase 4 inhibitor B) is a potent inhibitor of various tumors, which can inhibit the TGF‐β‐mediated cell cycle, and was also enriched in the TGF‐β signaling pathway of the hub genes. A clinical study showed that two SNPs (rs2383207 and rs2107595) of CDKN2B were significantly associated with the occurrence of IS (Akinyemi et al., 2017, 2018), which showed that CDKN2B was closely linked to the occurrence and progression of IS. And an another clinical study showed a positive correlation between the methylation level of CDKN2B gene and carotid artery calcification (Zhou et al., 2016). Carotid artery calcification is an important risk factor for the occurrence of IS. In an experimental study on stem cells, the activation of the TGF‐β signaling pathway was found to increase the expression of CDKN2B, which in turn promoted the endothelial cells' differentiation from stem cells, thereby reducing the rate of proliferation of endothelial cells (Bai et al., 2017). Combining the GSVA and GSEA analysis in this study, low expression of CDKN2B after the occurrence of IS would activate the cellular senescence signaling pathway. In this regard, we can speculate that CDKN2B may activate the cell aging pathway at a certain threshold; when the expression of CDKN2B exceeds this threshold, the activation of the cell aging signaling pathway would be turned off. Alternately, due to differences in the personal constitution and sample size, some patients were not sensitive to the expression of CDKN2B. So, the prognosis of these people may be better. According to this, CDKN2B could have a certain prognostic value, which is worthy of further study.

Through a cross‐sectional study of MEN1 (Menin) the mutation of this gene was found to be associated with the occurrence of Cushing disease (Makri et al., 2018), and Cushing disease manifests as high cortisol levels. In this study, MEN1 was negatively correlated with the level of urinary cortisol. At present, there is no relevant research to confirm how MEN1 is involved in the regulation of urinary cortisol levels. However, case reports (Chen et al., 2022) and other studies (Vaduva et al., 2020) have suggested that MEN1 had a certain correlation with cortisol levels. Further research may bring varying discoveries.

After the verification of the two data sets, our results suggest that the four hub genes have high diagnostic value, and the diagnostic model constructed with hub genes is comprehensive. ROC, DCA, and calibration data suggest the diagnostic efficiency of the model to be high. With further clinical verification, these models could be applied in regular clinical practice.

In conclusion, four cortisol‐related hub biomarkers were excavated in this study. In combination with existing literature, their possible mechanisms in the incidence and progression of the disease were explored. At the same time, there are certain limitations to the results of this study. It has not conducted relevant gene function research. But we have innovatively studied the molecular mechanism between cortisol and IS. The potential regulatory drugs and transcription factors were identified, and finally, a biomarker that is suitable for clinical use was proposed, providing novel insights into the key change of cortisol during the manifestation of IS.

## AUTHOR CONTRIBUTIONS

WXZ and XYM contributed to the searched the literature and designed the study; WJJ and XFB contributed to the acquisition of data; HS and XFB assisted in the data processing and performed the functional analysis; WJJ reviewed the data and drafted the manuscript. All authors have read and approved the final manuscript.

## CONFLICT OF INTEREST STATEMENT

The authors declare that they have no competing interests.

### PEER REVIEW

The peer review history for this article is available at https://publons.com/publon/10.1002/brb3.3358.

## Data Availability

GSE16561 (https://www.ncbi.nlm.nih.gov/geo/query/acc.cgi?acc = GSE16561) and GSE58294 (https://www.ncbi.nlm.nih.gov/geo/query/acc.cgi?acc = GSE58291) datasets used and/or analyzed during the current study are available from the corresponding author on reasonable request.

## References

[brb33358-bib-0001] Akinyemi, R. , Arnett, D. K. , Tiwari, H. K. , Ovbiagele, B. , Sarfo, F. , Srinivasasainagendra, V. , Irvin, M. R. , Adeoye, A. , Perry, R. T. , Akpalu, A. , Jenkins, C. , Owolabi, L. , Obiako, R. , Wahab, K. , Sanya, E. , Komolafe, M. , Fawale, M. , Adebayo, P. , Osaigbovo, G. , … Owolabi, M. (2017). Interleukin‐6 (IL‐6) rs1800796 and cyclin dependent kinase inhibitor (CDKN2A/CDKN2B) rs2383207 are associated with ischemic stroke in indigenous West African Men. Journal of The Neurological Sciences, 379, 229–235. 10.1016/j.jns.2017.05.046 28716248 PMC5546618

[brb33358-bib-0002] Akinyemi, R. , Tiwari, H. K. , Arnett, D. K. , Ovbiagele, B. , Irvin, M. R. , Wahab, K. , Sarfo, F. , Srinivasasainagendra, V. , Adeoye, A. , Perry, R. T. , Akpalu, A. , Jenkins, C. , Arulogun, O. , Gebregziabher, M. , Owolabi, L. , Obiako, R. , Sanya, E. , Komolafe, M. , Fawale, M. , … Owolabi, M. (2018). APOL1, CDKN2A/CDKN2B, and HDAC9 polymorphisms and small vessel ischemic stroke. Acta Neurologica Scandinavica, 137(1), 133–141. 10.1111/ane.12847 28975602 PMC5716854

[brb33358-bib-0003] Albani, A. , Pérez‐Rivas, L. G. , Dimopoulou, C. , Zopp, S. , Colón‐Bolea, P. , Roeber, S. , Honegger, J. , Flitsch, J. , Rachinger, W. , Buchfelder, M. , Stalla, G. K. , Herms, J. , Reincke, M. , & Theodoropoulou, M. (2018). The USP8 mutational status may predict long‐term remission in patients with Cushing's disease. Clinical Endocrinology, 89, 454–458. 10.1111/cen.13802 29957855

[brb33358-bib-0004] Aresta, C. , Favero, V. , Morelli, V. , Giovanelli, L. , Parazzoli, C. , Falchetti, A. , Pugliese, F. , Gennari, L. , Vescini, F. , Salcuni, A. , Scillitani, A. , Persani, L. , & Chiodini, I. (2021). Cardiovascular complications of mild autonomous cortisol secretion. Best Practice & Research. Clinical Endocrinology & Metabolism, 35(2), 101494. 10.1016/j.beem.2021.101494 33814301

[brb33358-bib-0005] Badawi, A. F. , Cavalieri, E. L. , & Rogan, E. G. (2001). Role of human cytochrome P450 1A1, 1A2, 1B1, and 3A4 in the 2‐, 4‐, and 16alpha‐hydroxylation of 17beta‐estradiol. Metabolism‐Clinical and Experimental, 50(9), 001–1003. 10.1053/meta.2001.25592 11555828

[brb33358-bib-0006] Bai, H. , Gao, Y. , Hoyle, D. L. , Cheng, T. , & Wang, Z. Z. (2017). Suppression of transforming growth factor‐β signaling delays cellular senescence and preserves the function of endothelial cells derived from human pluripotent stem cells. Stem Cells Translational Medicine, 6(2), 589–600. 10.5966/sctm.2016-0089 28191769 PMC5442820

[brb33358-bib-0007] Barugh, A. J. , Gray, P. , Shenkin, S. D. , Maclullich, A. M. J. , & Mead, G. E. (2014). Cortisol levels and the severity and outcomes of acute stroke: A systematic review. Journal of Neurology, 261(3), 533–545. 10.1007/s00415-013-7231-5 24477489 PMC4928702

[brb33358-bib-0008] Bolstad, B. M. , Irizarry, R. A. , Astrand, M. , & Speed, T. P. (2003). A comparison of normalization methods for high density oligonucleotide array data based on variance and bias. Bioinformatics (Oxford, England), 19, 185–193. 12538238 10.1093/bioinformatics/19.2.185

[brb33358-bib-0009] Wissler, C. (1905). The Spearman correlation formula. Science, 22(558), 309–311. 17836577 10.1126/science.22.558.309

[brb33358-bib-0010] Chen, F. , Xu, Q. , Yue, W. , Yu, X. , & Shao, S. (2022). A MEN1 patient presenting with multiple parathyroid adenomas and transient hypercortisolism: A case report and literature review. Frontiers in Endocrinology, 13, 802453. 10.3389/fendo.2022.802453 35370956 PMC8965320

[brb33358-bib-0011] Chen, H. , & Boutros, P. C. (2011). VennDiagram: A package for the generation of highly‐customizable Venn and Euler diagrams in R. BMC Bioinformatics [Electronic Resource], 12, 35. 10.1186/1471-2105-12-35 21269502 PMC3041657

[brb33358-bib-0012] Chen, X.‐G. , Shi, S.‐Y. , Hu, L. , Chen, Y. , Sun, H.‐W. , Zhou, L. , Lu, Z.‐B. , Wang, H. , Wang, X.‐S. , Yu, J. , Zhao, Y. u‐J. , Lu, Y.‐M. , & Ye, J. (2022). Longitudinal changes in the hypothalamic‐pituitary‐adrenal axis and sympathetic nervous system are related to the prognosis of stroke. Frontiers in Neurology, 13, 946593. 10.3389/fneur.2022.946593 35968302 PMC9364825

[brb33358-bib-0013] Csardi, G. , & Tamas, N. (2006). The igraph software package for complex network research. InterJournal, complex systems 1695, 1–9.

[brb33358-bib-0014] Durcan, T. M. , & Fon, E. A. (2015). USP8 and PARK2/parkin‐mediated mitophagy. Autophagy, 11(2), 428–429. 10.1080/15548627.2015.1009794 25700639 PMC4502724

[brb33358-bib-0015] Freshour, S. L. , Kiwala, S. , Cotto, K. C. , Coffman, A. C. , Mcmichael, J. F. , Song, J. J. , Griffith, M. , Griffith, O. L. , & Wagner, A. H. (2021). Integration of the Drug‐Gene Interaction Database (DGIdb 4.0) with open crowdsource efforts. Nucleic Acids Research, 49(D1), D1144–D1151. 10.1093/nar/gkaa1084 33237278 PMC7778926

[brb33358-bib-0016] Han, H. , Cho, J.‐W. , Lee, S. , Yun, A. , Kim, H. , Bae, D. , Yang, S. , Kim, C. Y. , Lee, M. , Kim, E. , Lee, S. , Kang, B. , Jeong, D. , Kim, Y. , Jeon, H.‐N. , Jung, H. , Nam, S. , Chung, M. , Kim, J.‐H. , & Lee, I. (2018). TRRUST v2: An expanded reference database of human and mouse transcriptional regulatory interactions. Nucleic Acids Research, 46(D1), D380–D386. 10.1093/nar/gkx1013 29087512 PMC5753191

[brb33358-bib-0017] Hänzelmann, S. , Castelo, R. , & Guinney, J. (2013). GSVA: Gene set variation analysis for microarray and RNA‐seq data. BMC Bioinformatics [Electronic Resource], 14, 7. 10.1186/1471-2105-14-7 23323831 PMC3618321

[brb33358-bib-0018] Herpich, F. , & Rincon, F. (2020). Management of acute ischemic stroke. Critical Care Medicine, 48(11), 1654–1663. 10.1097/CCM.0000000000004597 32947473 PMC7540624

[brb33358-bib-0019] Jin, S. , Gao, J. , Qi, Y. , Hao, Y. , Li, X. , Liu, Q. , Liu, J. , Liu, D. , Zhu, L. , & Lin, B. (2020). TGF‐β1 fucosylation enhances the autophagy and mitophagy via PI3K/Akt and Ras‐Raf‐MEK‐ERK in ovarian carcinoma. Biochemical and Biophysical Research Communications, 524(4), 970–976. 10.1016/j.bbrc.2020.02.028 32059847

[brb33358-bib-0020] Lin, W. , Shen, P. , Song, Y. , Huang, Y. , & Tu, S. (2021). Reactive oxygen species in autoimmune cells: Function, differentiation, and metabolism. Frontiers in immunology, 12, 635021. 10.3389/fimmu.2021.635021 33717180 PMC7946999

[brb33358-bib-0021] Makri, A. , Bonella, M. B. , Keil, M. F. , Hernandez‐Ramirez, L. , Paluch, G. , Tirosh, A. , Saldarriaga, C. , Chittiboina, P. , Marx, S. J. , Stratakis, C. A. , & Lodish, M. (2018). Children with MEN1 gene mutations may present first (and at a young age) with Cushing disease. Clinical Endocrinology, 89(4), 437–443. 10.1111/cen.13796 29927501 PMC6341462

[brb33358-bib-0022] Mesaros, C. , Lee, S. H. , & Blair, I. A. (2010). Analysis of epoxyeicosatrienoic acids by chiral liquid chromatography/electron capture atmospheric pressure chemical ionization mass spectrometry using [13C]‐analog internal standards. Rapid Communications in Mass Spectrometry, 24(22), 3237–3247. 10.1002/rcm.4760 20972997 PMC3348553

[brb33358-bib-0023] Miró‐Mur, F. , Laredo, C. , Renú, A. , Rudilosso, S. , Zhao, Y. , Amaro, S. , Llull, L. , Urra, X. , Planas, A. M. , & Chamorro, Á. (2018). Adrenal hormones and circulating leukocyte subtypes in stroke patients treated with reperfusion therapy. Brain Behavior and Immunity, 70, 346–353. 10.1016/j.bbi.2018.03.018 29548995

[brb33358-bib-0024] Morgan, M. F. (2022). GSEABase: Gene set enrichment data structures and methods. R package version.

[brb33358-bib-0025] Newman, A. M. , Liu, C. L. , Green, M. R. , Gentles, A. J. , Feng, W. , Xu, Y. , Hoang, C. D. , Diehn, M. , & Alizadeh, A. A. (2015). Robust enumeration of cell subsets from tissue expression profiles. Nature Methods., 12(5), 453–457. 10.1038/nmeth.3337 25822800 PMC4739640

[brb33358-bib-0026] Olsson, D. r. T. (1990). Urinary free cortisol excretion shortly after ischaemic stroke. Journal of Internal Medicine, 228(2), 177–181. 10.1111/j.1365-2796.1990.tb00213.x 2394969

[brb33358-bib-0027] Ritchie, M. E. , Phipson, B. , Wu, D. i. , Hu, Y. , Law, C. W. , Shi, W. , & Smyth, G. K. (2015). limma powers differential expression analyses for RNA‐sequencing and microarray studies. Nucleic Acids Research, 43(7), e47. 10.1093/nar/gkv007 25605792 PMC4402510

[brb33358-bib-0028] Robin, X. , Turck, N. , Hainard, A. , Tiberti, N. , Lisacek, F. , Sanchez, J. C. , & Müller, M. (2011). pROC: An open‐source package for R and S+ to analyze and compare ROC curves. BMC Bioinformatics [Electronic Resource], 12, 77. 21414208 10.1186/1471-2105-12-77 PMC3068975

[brb33358-bib-0029] Saini, J. S. , Corneo, B. , Miller, J. D. , Kiehl, T. R. , Wang, Q. , Boles, N. C. , Blenkinsop, T. A. , Stern, J. H. , & Temple, S. (2017). Nicotinamide ameliorates disease phenotypes in a human iPSC model of age‐related macular degeneration. Cell Stem Cell, 20(5), 635–647.e7. 10.1016/j.stem.2016.12.015 28132833 PMC5419856

[brb33358-bib-0030] Shannon, P. , Markiel, A. , Ozier, O. , Baliga, N. S. , Wang, J. T. , Ramage, D. , Amin, N. , Schwikowski, B. , & Ideker, T. (2003). Cytoscape: A software environment for integrated models of biomolecular interaction networks. Genome Research, 13(11), 2498–2504. 10.1101/gr.1239303 14597658 PMC403769

[brb33358-bib-0031] Subramanian, A. , Tamayo, P. , Mootha, V. K. , Mukherjee, S. , Ebert, B. L. , Gillette, M. A. , Paulovich, A. , Pomeroy, S. L. , Golub, T. R. , Lander, E. S. , & Mesirov, J. P. (2005). Gene set enrichment analysis: A knowledge‐based approach for interpreting genome‐wide expression profiles. Proceedings of the National Academy of Sciences of the United States of America, 102(43), 15545–15550. 10.1073/pnas.0506580102 16199517 PMC1239896

[brb33358-bib-0032] Tanzi, P. , Cain, K. , Kalil, A. , Zierath, D. , Savos, A. , Gee, J. M. , Shibata, D. , Hadwin, J. , Carter, K. , & Becker, K. (2011). Post‐stroke infection: A role for IL‐1ra. Neurocritical Care, 14(2), 244–252. 10.1007/s12028-010-9490-7 21174170 PMC4096896

[brb33358-bib-0033] Tofovic, S. P. (2010). Estrogens and development of pulmonary hypertension: Interaction of estradiol metabolism and pulmonary vascular disease. Journal of Cardiovascular Pharmacology, 56(6), 696–708. 10.1097/FJC.0b013e3181f9ea8d 20881610 PMC3027839

[brb33358-bib-0034] Tsuchiya, Y. (2003). Critical enhancer region to which AhR/ARNT and Sp1 bind in the human CYP1B1 gene. Journal of Biochemistry, 133(5), 583–592. 10.1093/jb/mvg075 12801909

[brb33358-bib-0035] Vaduva, P. , Bonnet, F. , & Bertherat, J. (2020). Molecular basis of primary aldosteronism and adrenal cushing syndrome. Journal of the Endocrine Society, 4(9), bvaa075. 10.1210/jendso/bvaa075 32783015 PMC7412855

[brb33358-bib-0036] Villanueva, R. A. M. , & Chen, Z. J. (2019). ggplot2: Elegant graphics for data analysis (2nd ed.). Measurement: Interdisciplinary Research and Perspectives, 17(3), 160–167.

[brb33358-bib-0037] Wang, J. , Guan, Q. , Sheng, Y. , Yang, Y. i. , Guo, L. i. , Li, W. , Gu, Y. , & Han, C. (2021). The potential predictive value of salivary cortisol on the occurrence of secondary cognitive impairment after ischemic stroke. Neurosurgical Review, 44(2), 1103–1108. 10.1007/s10143-020-01256-9 32314117

[brb33358-bib-0038] Wang, Y. , Li, Z. , Gu, H. , Zhai, Y. , Zhou, Q. , Jiang, Y. , Zhao, X. Q. , Wang, Y. L. , Yang, X. , Wang, C. J. , Meng, X. , Li, H. , Liu, L. P. , Jing, J. , Wu, J. , Xu, A. D. , Dong, Q. , Wang, D. , Wang, W. Z. , … China Stroke Statistics Writing Committee . (2022). China Stroke Statistics: An update on the 2019 report from the National Center for Healthcare Quality Management in Neurological Diseases, China National Clinical Research Center for Neurological Diseases, the Chinese Stroke Association, National Center for Chronic and Non‐communicable Disease Control and Prevention, Chinese Center for Disease Control and Prevention and Institute for Global Neuroscience and Stroke Collaborations. Stroke and Vascular Neurology, 7(5), 415–450. 35443985 10.1136/svn-2021-001374 PMC9614174

[brb33358-bib-0039] Warde‐Farley, D. , Donaldson, S. L. , Comes, O. , Zuberi, K. , Badrawi, R. , Chao, P. , Franz, M. , Grouios, C. , Kazi, F. , Lopes, C. T. , Maitland, A. , Mostafavi, S. , Montojo, J. , Shao, Q. , Wright, G. , Bader, G. D. , & Morris, Q. (2010). The GeneMANIA prediction server: Biological network integration for gene prioritization and predicting gene function. Nucleic Acids Research, 38(Web Server issue), W214–W220. 10.1093/nar/gkq537 20576703 PMC2896186

[brb33358-bib-0040] Yu, G. , Wang, L.‐G. , Han, Y. , & He, Q.‐Y. (2012). clusterProfiler: An R package for comparing biological themes among gene clusters. Omics—A Journal of Integrative Biology, 16(5), 284–287. 10.1089/omi.2011.0118 22455463 PMC3339379

[brb33358-bib-0041] Zhang, B. , Subramanian, S. , Dziennis, S. , Jia, J. , Uchida, M. , Akiyoshi, K. , Migliati, E. , Lewis, A. D. , Vandenbark, A. A. , Offner, H. , & Hurn, P. D. (2010). Estradiol and G1 reduce infarct size and improve immunosuppression after experimental stroke. Journal of Immunology (Baltimore, Md.: 1950), 184, 4087–4094. 20304826 10.4049/jimmunol.0902339 PMC3142781

[brb33358-bib-0042] Zhang, F. , Fu, C. , Deng, Y. , Zhang, M. , Peng, H. , Li, W. , Zhong, J. , Zhou, Q. , Huang, L. i. , Xiao, S. , & Zhao, J. (2023). CYP1B1 genetic variants (rs10916 and rs2855658) are associated with increased risk of ischemic stroke in Chinese Han population. Cerebrovascular diseases (Basel, Switzerland), 52(3), 293–305. 10.1159/000526918 36634630

[brb33358-bib-0043] Zhao, J. , Bi, W. , Zhang, J. , Xiao, S. , Zhou, R. , Tsang, C. K. , Lu, D. , & Zhu, L. (2020). USP8 protects against lipopolysaccharide‐induced cognitive and motor deficits by modulating microglia phenotypes through TLR4/MyD88/NF‐κB signaling pathway in mice. Brain, Behavior, and Immunity, 88, 582–596. 10.1016/j.bbi.2020.04.052 32335193

[brb33358-bib-0044] Zhou, S. , Zhang, Y. , Wang, L. , Zhang, Z. , Cai, B. , Liu, K. , Zhang, H. , Dai, M. , Sun, L. , Xu, X. , Cai, H. , Liu, X. , Lu, G. , & Xu, G. (2016). CDKN2B methylation is associated with carotid artery calcification in ischemic stroke patients. Journal of Translational Medicine, 14(1), 333. 10.1186/s12967-016-1093-4 27905995 PMC5134267

[brb33358-bib-0045] Zierath, D. , Tanzi, P. , Shibata, D. , & Becker, K. J. (2018). Cortisol is more important than metanephrines in driving changes in leukocyte counts after stroke. Journal of Stroke & Cerebrovascular Diseases, 27(3), 555–562. 29097060 10.1016/j.jstrokecerebrovasdis.2017.09.048 PMC5811368

